# A field survey on parasites and antibodies against selected pathogens in owned dogs in Lilongwe, Malawi

**DOI:** 10.4102/jsava.v87i1.1358

**Published:** 2016-07-29

**Authors:** Karin Alvåsen, Sandra M. Johansson, Johan Höglund, Richard Ssuna, Ulf Emanuelson

**Affiliations:** 1 Department of Clinical Sciences, Swedish University of Agricultural Sciences, Sweden; 2 Department of Biomedical Sciences and Veterinary Public Health, Swedish University of Agricultural Science, Sweden; 3 Lilongwe Society for Protection and Care of Animals, Lilongwe, Malawi

## Abstract

The aim of this study was to screen for selected parasites and antibody levels against vector-borne pathogens in owned dogs in Lilongwe, Malawi. The study population consisted of 100 dogs; 80 participating in vaccination–spaying campaigns and 20 visiting a veterinary clinic as paying clients. All dogs went through a general physical examination including visual examination for signs of ectoparasites. A total of 100 blood samples were analysed using commercial snap tests and 40 faecal samples by egg flotation in saturated sodium chloride. The sampled dogs had a seroprevalence of 12% for *Anaplasma* spp., 22% for *Ehrlichia* spp., 4% for *Dirofilaria immitis* and 1% for *Leishmania* spp. Eggs from *Ancylostoma* spp. were found in 80% of the faecal samples, whereas eggs of *Trichuris vulpis, Toxocara canis* and *Toxascaris leonina* were only present in 3%, 8% and 13% of the samples, respectively. Ectoparasites such as *Ctenocephalides* sp., *Trichodectes* sp. and ticks were present on 98%, 25% and 11%, respectively, of the campaign dogs. Among client dogs, 35% had *Ctenocephalides* fleas, 10% had *Trichodectes* lice and none had ticks. Public education and prophylactic treatment could be used to improve the animal welfare of dogs; this would most likely also have positive impact on public health.

## Introduction

According to the concept of ‘One Health’, improvement of animal health contributes to the health of humans. Diseases in the animal population may constitute a threat to public health (Lavallén *et al*. 2011; Matjila *et al*. 2008; Schurer *et al*. 2013), especially in low income countries (Bwalya *et al*. 2011; Esemu, Ndip & Ndip 2011; Sowemimo & Asaolu 2008) such as Malawi.

In Lilongwe, the capital of Malawi, the human population reached 905 000 in 2015 (CIA 2015) and the dog population was approximately 100 000 (of which 36 500 were strays) in 2013 (Boone 2013). The dog is a domestic animal that lives in close contact with humans and other animals. Despite their beneficial effects, dogs are associated with many zoonotic diseases and pose public health concerns worldwide (Millán *et al*. 2013; Reaser, Clark & Meyers 2008; Slater 2001; Yabsley *et al*. 2008). To prevent the spread of animal diseases and zoonotic pathogens, it is necessary to establish which pathogens are present (Irwin 2014; Noden & Soni 2015).

With the exception of rabies, there is a dearth of information on the epidemiology of canine pathogens in Malawi. To our knowledge, there is only one study (Fitzsimmons 1967) on parasites and other microbes within the dog population in southern Malawi. The objective of the present field study was to screen for the presence of selected parasites and antibody levels against selected vector-borne pathogens in the dog population in Lilongwe, Malawi.

## Methods

### Study site and selection of dogs

This study was carried out in the urban and peri-urban areas of Lilongwe, Malawi, during September and October 2014. It was performed in accordance with local guidelines for non-experimental research of the Lilongwe Society for Protection and Care of Animals (LSPCA) and Department of Clinical Sciences, Swedish University of Agricultural Sciences (SLU). Owned dogs participating in rabies vaccination and spaying campaigns and dogs visiting a veterinary clinic were eligible for inclusion, and ethical approval for such non-experimental research was not needed. The vaccination-spaying campaigns and the veterinary clinic are both run by LSPCA. The rabies vaccination campaign is conducted for 2 weeks every autumn, and in 2014 it included approximately 16 000 dogs. The spaying campaign runs all-year round, twice-weekly. The dogs participating in campaigns were either free-roaming or kept confined outdoors. Areas included in this study were initially selected randomly. Later specific areas were targeted to ensure that free-roaming and confined dogs were equally represented. A total of 80 campaign dogs (40 free-roaming and 40 confined) were included in the study. In addition, 20 dogs visiting the LSPCA clinic as paying clients (hereafter referred to as client dogs) were included. Client dogs came from the urban areas and were generally kept indoors and/or fenced-in. Participating dogs were brought to either the campaign or to the clinic by the owner. The owner was informed about the purpose of the present study and gave permission to collect and use samples.

### Questionnaire

Dog owners filled out a questionnaire in either English or the local language Chichewa. The questionnaire covered aspects such as the dog’s age, how it was kept, veterinary visits, vaccinations and use of endo- and/or ectoparasiticides.

### Sample collection

All dogs enrolled went through a physical examination, which included evaluation of their weight, mucosal inspection and palpation of the lymph nodes. Blood (~5 mL) from the *vena cephalica* was added to Ethylenediamine tetraacetic acid (EDTA) vacutainer tubes. Blood was either immediately transferred to a snap test or transported in a freezer box with a cool pack to the laboratory of LSPCA to be analysed within 24 h.

Faecal samples were collected from the rectum of half of the campaign dogs (40 out of 80). It was not possible to sample all dogs as some were stressed or did not have enough faeces at the time of sampling. Faeces were collected both from dogs being awake and from dogs under anaesthesia. Oil was used as a lubricant when needed. Nitrile gloves were used during the collection, and the faeces was put in a 10-mL plastic transport tube, transported in a freezer box with a cool pack, for analysis at the laboratory of LSPCA within 24 h.

### Sample analysis

#### Visual analysis

The coat of the dogs was visually examined for presence and signs of ectoparasites. Special attention was given to the ears since these are one of the predilection sites for ticks (Jacobs *et al*. 2001).

#### Faecal analysis

Faecal analysis for presence of helminth eggs was done by egg flotation. Fresh faeces (~5 mL) were placed in a 10-mL transport tube before adding tap water until the tube was almost full. The contents were mixed to a homogenous solution that was sieved through a 150 µm mesh into a centrifuge tube and centrifuged (for 10 min at 1286 g). The supernatant was discarded and the faecal pellet resuspended in 5 mL saturated NaCl, mixed, and then topped up with an additional 5 mL. Eggs were collected by floating a coverslip on the surface of the resuspended pellet for at least 15 min. The coverslip was thereafter transferred to a glass slide and examined microscopically at 100× and 400× magnification. Eggs were identified by using their morphological characteristics (Taylor, Coop & Wall 2007); dogs were classified as parasite positive when a helminth egg was observed.

#### Serological analysis

The blood was analysed for circulating antibodies against *Anaplasma* spp.*, Borrelia burgdorferi, Dirofilaria immitis, Ehrlichia* spp. and *Leishmania* spp. Two commercial ELISA (Enzyme-Linked ImmunoSorbent Assay) tests were used: Idexx SNAP^®^ 4Dx^®^ Plus (IDEXX Laboratories, Inc., Westbrook, United States) and BVT Speed Leish K (BVT, La Seyne sur Mer, France).

### Statistical analysis

Fisher’s exact test was used to compare differences between dog populations regarding prevalence of selected parasites and antibodies. *p*-value < 0.05 was considered statistically significant. A binominal exact confidence interval with a 95% confidence level was calculated for the prevalences using the Clopper-Pearson (exact) method (Danielsoper 2015).

## Results

### Questionnaire

The campaign dogs were 0.5–13 years old with an average age of 3.8 years (median 3.0 years). The client dogs were between 0.6 and 13 years old with an average age of 4.1 years (median 3.5 years).

Forty-one percent (33 out of 80) of the campaign dogs and 85% (17 out of 20) of the client dogs had visited a veterinarian at least once. Of the campaign dogs, 70% (56 out of 80) had been vaccinated against rabies and one of these dogs had also been vaccinated against parvo virus. Of the client dogs, 95% (19 out of 20) had been vaccinated against rabies and 80% (16 out of 20) against parvo virus. Of the campaign dogs 31% (25 out of 80) had been dewormed at some point, while 80% (16 out of 20) of the client dogs were dewormed regularly.

Of the campaign dogs, 55% (44 out of 80) received ectoparasiticides once every 1–3 months. Some communities dipped all the village dogs in amitraz every 1–2 months. Eighteen client dog owners (90%) reported that they treated their dogs against ectoparasites monthly, usually with an antiparasitic shampoo containing pyrethrin, and one owner used spot on products (fipronil). Tick collars or other substances against ectoparasites were not used.

### Physical examination

#### General health status

Most dogs were in good body condition, but 35% of client dogs were moderately overweight and 15% of campaign dogs were underweight. Two dogs (one confined campaign dog and one client dog) had clinical signs of anaemia.

#### Ectoparasites

Visual examination of the dogs’ coats showed a high prevalence of *Ctenocephalides* sp. (Table 1), but species was not defined. The prevalence of *Ctenocephalides* fleas was significantly higher among campaign dogs than in client dogs, but there was no difference between the two campaign groups (free-roaming vs. confined). Many dogs had wounds on their scalps and outer ears, likely because of fly bites. Ticks were only found on campaign dogs. Of the nine campaign dogs with ticks, eight were free-roaming of which seven were also positive for antibodies against *Ehrlichia* spp. There was no statistically significant difference between campaign and client dogs concerning prevalence of lice and ticks (Table 1).

**TABLE 1:** Prevalence (with confidence intervals) of ectoparasites in dogs in Lilongwe, Malawi, September–October 2014.

Type of dog	Fleas		Lice		Ticks	
		
	*n* positive	Prevalence (95% CI)	*n* positive	Prevalence (95% CI)	*n* positive	Prevalence (95% CI)
Campaign (*n* = 80)	78	97.5 (88.9; 99.9)	20	25.0 (16.0; 35.9)	9	11.3 (5.3; 20.3)
Free-roaming (*n* = 40)	39	97.5 (86.8; 99.9)	13	32.5 (18.6; 49.1)	8	20.0 (9.1; 35.6)
Confined (*n* = 40)	39	97.5 (86.6; 99.9)	7	17.5 (7.3, 32.8)	1	2.5 (0; 13.2)
Client (*n* = 20)	7	35.0 (15.4; 59.2)	2	10.0 (1.2; 31.7)	0	0.0 (0; 16.8)
**Total (*n* = 100)**	**85**	**85.0 (78.0; 92.0)**	**22**	**22.0 (14.3; 31.4)**	**9**	**9.0 (4.2; 16.4)**

#### Endoparasites

Hookworm eggs (*Ancylostoma* sp.) were present in 80% (32 out of 40) of the faecal samples (Table 2). Eggs of other genera sporadically identified were *Trichuris, Toxocara* and *Toxascaris*.

**TABLE 2:** Prevalence (with confidence intervals) of endoparasites in faeces from 40 campaign dogs in Lilongwe, Malawi, September–October 2014.

Genus	*n* positive	Prevalence (95% CI)
*Ancylostoma* spp.	32	80.0 (64.4; 90.9)
*Trichuris vulpis*	1	2.5 (0.0; 13.2)
*Toxocara canis*	3	7.5 (1.6; 20.1)
*Toxoscaris leonina*	5	12.5 (4.2; 26.8)

### Serology

Twelve dogs were seropositive for *Anaplasma* spp. (Table 3) and four of these dogs also had ticks. There was no statistically significant difference in seroprevalence between free-roaming and confined campaign dogs, or between campaign and client dogs.

**TABLE 3:** Prevalence (with confidence intervals) of serum antibodies against infectious agents in dogs in Lilongwe, Malawi, September–October 2014.

Type of dog	*Anaplasma* spp.		*Ehrlichia* spp.	
	
*n* positive	Prevalence (95% CI)	*n* positive	Prevalence (95% CI)
Campaign (*n* = 80)	11	13.8 (7.1; 23.3)	21	26.3 (14.7; 40.7)
Free-roaming (*n* = 40)	8	20.0 (9.1; 35.6)	15	37.5 (22.7; 54.2)
Confined (*n* = 40)	3	7.5 (1.6; 20.4)	6	15.0 (5.7; 29.8)
Client (*n* = 20)	1	5.0 (0.1; 24.9)	1	5.0 (0.1; 24.9)

**Total (*n* = 100)**	**12**	**12.0 (6.4; 20.0)**	**22**	**22.0 (14.3; 31.4)**

One dog had antibodies against *Leishmania* spp. This dog was 1 year old, free-roaming and participated in the rabies vaccination campaign. Four dogs were seropositive for *D. immitis*. These four dogs were free-roaming and participated in the campaigns. Two of them were elderly (8–10 years old), one was middle-aged (exact age unknown) and one was 9 months old. No dog was seropositive for *B. burgdorferi*.

Antibodies against *Ehrlichia* spp. were found in 22% (22 out of 100) of the dogs (Table 3). There was no statistically significant difference in prevalence between campaign and client dogs. The only client dog seropositive for *Ehrlichia* spp. was free-roaming. The prevalence among the free-roaming campaign dogs was significantly higher than in the confined campaign dogs. Ticks were identified on 7 of the 21 campaign dogs that were seropositive for *Ehrlichia* spp.

## Discussion

### General health

The brief general examination found that 85% of the campaign dogs were in good condition and 15% were underweight. The latter could be a consequence of underfeeding but could also indicate chronic disease. Furthermore, free-roaming dogs in poor condition were probably less likely to be vaccinated or spayed and the general health status in this population could therefore have been overestimated. The average age was low (about 4 years) indicating a high turnover rate or that Lilongwe dogs are neutered at a young age.

### Parasites and antibodies against vector-borne pathogens

Ectoparasites were common with fleas being present on the majority of dogs. This result was expected as a high prevalence of fleas in dogs is common in several developing countries in the tropics (Colombo *et al*. 2011; Kumsa & Mekonnen 2011; Wells *et al*. 2012). The high density of free-roaming dogs provides ample opportunity for the transmission of ectoparasites and is possibly an explanation for the high prevalence found. Ectoparasiticides were infrequently used. Fleas were common in areas where dogs were dipped regularly, as amitraz is not effective on fleas (Folz *et al*. 1986). The low number of dogs infected with lice might be because lice are more difficult to detect by visual examination. The true prevalence of lice was most likely higher. The prevalence of dogs with ticks was also low in the present study. However, as antibodies to tick-borne pathogens *Anaplasma* spp. and *Ehrlichia* spp. were abundant in the study population, with *Ehrlichia* spp. in higher numbers, the prevalence of tick infestations could also have been underestimated. During a more thorough examination of 10 dogs that were under anaesthesia, but not part of the study, ticks were found in the ears of all dogs, further strengthening this hypothesis. The significant difference in prevalence of ticks between campaign and client dogs is probably because client dogs were treated more regularly against ectoparasites. Client dogs were also usually confined, which reduced the exposure to infested dogs and risk environments.

The heartworm *D. immitis* frequently occurs in tropical countries where the mosquito vectors are present throughout the year (Davoust *et al*. 2008). In the present study, only 4% of the dogs were seropositive for *D. immitis*. The low prevalence may be because of the young age of the study population, as the prevalence has been shown to increase with age (Vezzani *et al*. 2011), but may equally be because of absence of suitable vectors in the study area. None of the client dogs had antibodies against heartworm. Although there was no statistically significant difference in prevalence between the campaign and client populations, the risk of mosquito bites is likely to be reduced when dogs are kept indoors. A larger number of client dogs would be needed to confirm this interpretation.

Twelve percent of the dogs that were seropositive for *Anaplasma* spp. *Anaplasma phagocytophilum*, a species closely related to *Anaplasma platys*, have been documented in Africa and are all known to cross react with the ELISA test used. Polymerase chain reaction (PCR) will be necessary to identify *Anaplasma* species present in Malawi. No dog in the present survey was seropositive for *B. burgdorferi*, and this result was expected as *B. burgdorferi* has not yet been detected in Southern Africa (Gern & Falco 2000).

Over one-fifth (22%) of the dogs in the present survey were seropositive to *Ehrlichia* spp. antibodies. This seroprevalence is much lower than those detected in Tunisia, Mexico and Kenya, 54% (155 out of 286), 44% (53 out of 120) and 86% (56 out of 65), respectively (M’Ghirbi *et al*. 2009; Rodriguez-Vivas, Albornoz & Bolio 2005; Woodroffe *et al*. 2012). The seroprevalence among the free-roaming dogs (38%) in the present survey was however higher than that reported in rural dogs in Uganda (30%; Proboste *et al*. 2015) or in dogs in Maasai Mara, Kenya (16%; Alexander *et al*. 1993).

It is noteworthy that only one dog in this study was seropositive for *Leishmania* spp. This seropositive dog might be a false positive because *Leishmania* spp. are rarely seen in Malawi (WHO 2012). Thus, the prevalence of this zoonotic parasite seems to be low in owned dogs in the studied areas of Lilongwe, which is an important finding from a medical perspective (Ashford 2000; Greene 2006).

Most faecal samples (80%) contained eggs of the tropical hookworm *Ancylostoma* spp. This result is similar to the 88% reported from the Southern Province of Malawi (Fitzsimmons 1967), 79% reported from pet and stray dogs in northwest Ethiopia (Abere, Bogale & Melaku 2013) and 72% reported from Zambia (Bwalya *et al*. 2011), but higher than the 35% reported in Gabon (Davoust *et al*. 2008). These differences in prevalence may reflect differences in climate, sampling, veterinary facilities and public awareness (Abere *et al*. 2013). Malawi has a tropical climate, which allows this parasite to survive in soil for several weeks (Gasser, Cantavessi & Loukas 2008). *Ancylostoma* spp. may be transmitted from the environment by direct ingestion of larva as well as transcutaneously (Traub *et al*. 2014). Direct transmission from dam to pups may occur transplacentally or via her milk amplifying the parasite burden in the population (Bowman *et al*. 2010; Swai *et al*. 2010). The climate, the variety of transmission routes available to this parasite, the large population of stray dogs that are never dewormed and the absence of social pressure that convinces dog owners to pick up their dogs’ faeces facilitate a high environmental contamination with hookworm larvae.

In this survey, two species of ascarids, *Toxocara canis* and *Toxascaris leonine*, were found in 8% (3 out of 40) and 13% (5 out of 40) of the dogs. The prevalence of *T. leonina* in dogs is normally higher in older dogs than in puppies (Minnaar, Krecek & Fourie 2002), and the results from the present field study are in agreement with figures from South Africa (Minnaar & Krecek 2001). The proportion of dogs infested with *T. canis* in this study is lower than the reported prevalence of *T. canis* in Ethiopia (40%), and Gabon (58.5%; Abere *et al*. 2013; Davoust *et al*. 2008). In contrast, Fitzsimmons (1967) reported a total absence of ascarid worms in 120 euthanised Malawian dogs. As *T. canis* migrate to the mammary glands in adult dogs (Overgaauw & Van Knapen 2013; Rubinsky-Elefant *et al*. 2010), the true prevalence of Toxocara-infected dogs in Lilongwe was most likely underestimated. Puppies, which are the main egg shedders (Minnaar, Krecek & Rajput 1999), were not included in the present study. Dogs under 6 months of age were however included in the Ethiopian study (Abere *et al*. 2013), which partly explains the difference in prevalence between the two studies. A low number of dogs can pass a large number of eggs in their faeces, and *T. canis* eggs can survive in the environment for several years (Overgaauw & van Knapen 2013).

All three of the above worms may be zoonotic. Infection may occur by direct ingestion of eggs or larvae of all three species through direct or indirect contact (e.g. on dog’s coat or contaminated soil) with infected faeces. Cutaneous larval migrans may develop when Ancylostoma larva penetrate the skin (Bowman *et al*. 2010). Visceral larva migrans may develop after ingestion of Toxocara eggs (Amaral *et al*. 2010). This is much more common with Toxocara canis but has rarely been reported with Toxocara leonina. Children are at highest risk for exposure as they frequently handle puppies, play in the dirt and generally have lower hygiene standards than adults (Rubinsky-Elefant *et al*. 2010).

## Conclusion

The owned dog population in Lilongwe, Malawi, was exposed to pathogens that can cause diseases and poor welfare. This also poses health risks for humans. The relatively high prevalence of parasites and vector-borne pathogens, combined with the high number of dogs in Lilongwe, makes the disease pressure considerable. Introducing measures to control these pathogens would not only improve animal welfare but also contribute to improved public health, and they therefore merit serious consideration.
